# Prioritizing the perceived equity of the residents to construct an equitable health care system: evidence from a national cross-sectional study in China

**DOI:** 10.1186/s12913-020-5026-9

**Published:** 2020-03-04

**Authors:** Hui Lv, Jianqin Gu, Xiangdong Yuan, Yudong Miao

**Affiliations:** 1 Management Institute of Xinxiang Medical University, Xinxiang, China; 2 Department of General Medicine, Henan Provincial People’s Hospital, School of Clinical Medicine, Henan University, Zhengzhou, China; 3 Department of General Surgery of Guangdong General Hospital, Guangzhou, China; 4 Department of General Medicine, Henan Provincial People’s Hospital, People’s Hospital of Zhengzhou University, 7 Weiwu Road, Zhengzhou, 450003 Henan Province China

**Keywords:** Equity, Health care system, Perception

## Abstract

**Background:**

Building an equitable health care system involves both the promotion of social justice in health and people’s subjective perception of the promotion. This study aimed to analyze the overall status and associated factors of the perceived equity of the Chinese health care system, and then to offer policy recommendations for health care reform.

**Methods:**

Information on the perceived equity score (scale 0 to 10) of 10,243 valid cases in total were derived from the data set of Chinese Social Survey 2015. Univariate analysis methods were applied to present respondents’ overall perceived equity of the Chinese health care system. Multivariate linear regression method was used to explore the associated factors of the perceived equity and examine their independent effect.

**Results:**

The respondents gave positive but relatively low marks (6.7 ± 2.6, 95% CI: = 6.64~6.74) of the equity of the Chinese health care system. Younger respondents reported a higher score of perceived equity than their elder counterparts (β = − 0.132, 95% CI: − 0.203~ − 0.062, *P* < 0.001). Respondents with lower education level were significantly more likely to consider the Chinese health care system equitable (β = − 0.104, 95% CI: − 0.153~ − 0.056, *P* < 0.001). Respondents satisfied with the Social Health Insurance reimbursement ratio tended to score the system higher in the survey (β = 0.044, 95% CI: 0.024~0.063, *P* < 0.001). Respondents residing in eastern China and rural areas were significantly more likely to consider the Chinese health care system equitable (β = − 0.268, 95% CI: − 0.338~ − 0.199, *P* < 0.001). Meanwhile, rural respondents reported higher scores of the perceived equity than urban respondents did (β = 0.348, 95% CI: 0.237~0.458, *P* < 0.001). Respondents from regions with adequate GPs scored the system higher in this survey (β = 0.087, 95% CI: 0.008~0.165, *P* < 0.001). The present study found no influence of gender, economic status, Social Health Insurance coverage, or satisfaction with the latest treatment on perceived equity.

**Conclusions:**

Eliminating the sense of inequity among a range of populations should be prioritized in health care reform. A national-level investigation system to rate residents’ perceived equity was necessary for global health care reform.

## Background

In World Health Report 2000 - Health Systems: Performance Improving, the World Health Organization (WHO) defined health systems as “all the activities whose primary purpose is to promote, restore or maintain health” [1]. It proposed that a high-quality health care system should focus on and meet the health needs of different groups of people and would not cause health gaps between economic levels and the ability to pay [2]. Over the past decade after the presentation of the report, building an equitable health care system has become one of the core objectives of health care reform in most countries [3–5]. Building an equitable health care system involves both the promotion of social justice in health (i.e., no one is denied or discriminated to live a healthy life due to economically and socially deprived) [6] and people’s subjective perception of the promotion. However, for one thing, health care systems in many countries have been unable to introduce or sustain improvements in the equity of health outcomes [7], health service use [8], health financing [9, 10], health resource allocation [11], and so on. For another, researches and interventions that focus only on the technical, clinical, or financial dimensions of health interventions and systems generally lose sight of people’s subjective perception of the equity of the health care system in their country. Many policymakers have realized that the construction of an equitable health care system, as a public affair, should try not only to reshape the health care system itself but also to serve public interests and justice in the reformed health care system [12]. Perceived equity, more than equity in one aspect of a country’s health care system, should be monitored and taken as an important reference for the top-level design of health policy in terms of improving the equity of a country’s health care system.

**Fig. 1:**
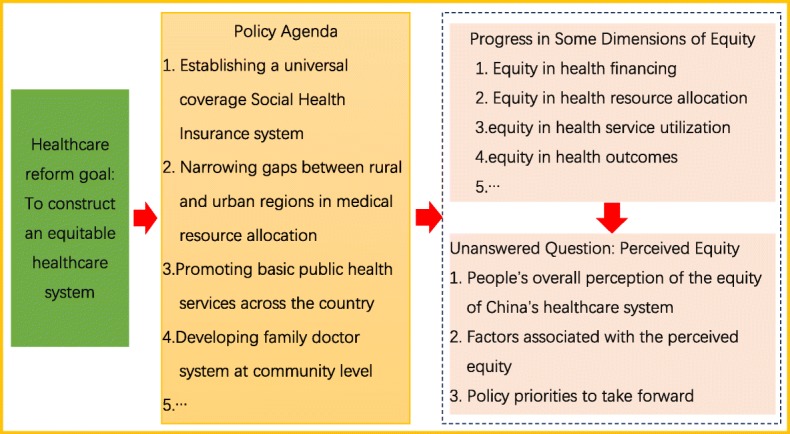
Equity in the Chinese health care system: policy agenda, progress, and unanswered question

As the most populous country, China’s health care reform affects global health, not only because the large population comprises a fifth of the world’s population, but also because its innovations and experiences will be helpful and influential for many low- and middle-income countries [13]. Through increasing investment in health care and strong policy intervention [14], China’s health care system has achieved some progress in ensuring equity, including by establishing a universal-coverage Social Health Insurance (SHI) system [15, 16], narrowing gaps between rural and urban regions in medical resource allocation [17], promoting basic public health services across the country [18], and developing a family doctor system at the community level [19]. These ongoing achievements, presaging an equitable health care system, are being set up. However, it should be noted that in a country as vast as China, the equity of a health care system is a complex and social topic for global health care reform. It requires policymakers to focus on the internal structure of health systems as well as on people’s perception of the equity of the health care system (see Fig. 1).

In this article, we define perceived equity to be residents’ subjective perception of the equity of the health care system in the country they are living in. We aim to present (1) residents’ overall perceived equity of the Chinese health care system, (2) associated factors influencing the perceived equity, and (3) policy orientation for global health care reform in terms of improving the perceived equity. These concerns are not only unique to China but also common challenges for global health care reform, affecting health policy design now and in the future.

## Methods

### Data source

The data of this study was derived from the dataset of Chinese Social Survey (CSS) 2015. As a comprehensive survey with the greatest impact in the field of social development, CSS was conducted in 2006, 2008, 2011, 2013, and 2015 by the Institute of Sociology of the Chinese Academy of Social Sciences. The sample size of each CSS was different. CSS 2015 adopted a multistage, stratified, random sampling method and covered 31 provinces, 151 counties, and 604 communities throughout the Chinese mainland. In total, CSS 2015 surveyed 10,268 respondents, who were aged between 18 to 70 and most familiar with their family conditions. CSS 2015 was conducted from May 2015 to November 2015. The detailed introduction of CSS 2015 was available at http://css.cssn.cn/css_sy/xmjs/ (in Chinese). Authors used the original dataset of CSS 2015 to measure respondents’ perceived equity of the Chinese health care system. Respondent’s perceived equity was analyzed on a 0–10 scale, 0 representing inequitable and 10 representing equitable. Meanwhile, authors also used the survey data to measure demographics, socioeconomic status, SHI coverage, inhabited regions and satisfaction with the latest treatment. In addition, authors added the number of general practitioners (GP’s) per 10,000 residents in 2015 into the data set according to the province of the respondents. The data about GPs were collected from the China Health and Family Planning Statistics Yearbook 2015. Authors were authorized to use academic data research by the CSS 2015 research team. Ten thousand two hundred forty-three valid cases were obtained after eliminating cases with incomplete information or logical errors.

### Respondent characteristics

This study used 10 socio-demographic variables as potential social determinants in the analysis. Among these variables, seven indicators were taken directly from the CSS 2015 questionnaire to perform analysis: gender (female as reference), education (illiteracy as reference), SHI coverage (not covered by SHI as reference), satisfaction of SHI reimbursement ratio (not satisfied as reference), geographical area (eastern area as reference), district (urban as reference), and satisfaction with the latest treatment (not satisfied as reference). Respondents were divided into three groups according to their age: a youth group (18 to 44, as reference), a middle-aged group (45 to 59), and an old-aged group (60 and above). Urban and rural respondents whose annual household incomes per capita were lower than 5400 Yuan and 2000 Yuan (according to the regulation of the Ministry of Civil Affairs of China), respectively, were categorized into a low-income family group (as reference). The rest families were categorized to be a not low-income family group. The number of general practitioners per 10,000 residents of each province, as an important indicator of medical resources, was collected through China’s Health and Family Planning Statistical Yearbook 2015 and added to the data set of CSS 2015. Accordingly, respondents were categorized into three groups based on the number of general practitioners per 10,000 residents in their inhabited province: inadequate group (last 25%, as reference), medium group (middle 50%), and adequate group (top 25%).

### Statistical analysis

Socio-demographic data were described for all valid respondents. Continuous variables were expressed as mean ± standard deviation and the difference between/among groups was compared using a *t*-test or one-way analysis of variance analysis (ANOVA). Categorical variables were expressed as percentages and differences between/among groups were tested using chi-square tests. To examine the independent effects of sex, age, education, economic status, SHI coverage, the satisfaction of SHI reimbursement ratio, geographical area, district, general practitioners per 10,000 residents, and the satisfaction with the latest treatment on perceived equity of health care system, the multivariate linear regression method was applied. The variable “the perceived equity score of the respondents” was set as the dependent variable of the multivariate linear regression. We used a stepwise selection method in the statistical analysis procedure. *P* < 0.05 was considered statistically significant. Statistical analyses were performed using SPSS version 22.0 for Windows (Chicago, IL, USA).

### Ethical issues

All enrolled respondents gave written informed consent to the CSS research team. The questionnaire and the dataset of CSS 2015 were anonymized. Not all responses could be traced to the respondents and respondents could not be identified. The data source for CSS is open-access, and the data set is available at http://css.cssn.cn/css_sy/zlysj/lnsj/. We were authorized for academic research after the application was approved through email on October 25, 2017.

### Patient and public involvement

No patients were directly involved in the recruitment and design of this cross-sectional study. Before the face-to-face survey, all participants were informed about the research questions and study objectives. The principal findings of this study were to be used in evidence-based health policy decision making to help China and other low- and middle- income countries construct a more equitable health care system.

## Results

### Demographics

Table 1 detailed the socio-demographic characteristics of 10,243 respondents who reported their perceived equity of the Chinese health care system. Of the respondents, 54.6% (*n* = 5592) were female and 45.4% (*n* = 4651) were male. The majority of respondents were younger than 60 (78.1%) and had low family income (74.6%). 72.5% of the respondents came from areas with inadequate GPs. When examining the education background, 11.8% of the respondents were illiterate and 14.6% held a bachelor degree or higher. A total of 91.1% of the respondents were covered by Chinese SHIs and the rest (8.9%) were still not covered. Approximately 30% of the respondents were satisfied with the SHI reimbursement ratio (29.5%) and their latest use of medical services (28.7%). In terms of the regional geographic distribution of the respondents, the proportions of eastern, central, and western areas were 41.5, 31.6, and 26.9%, respectively. More than half of the respondents resided in an urban area (54.5%).

**Table 1:** Demographics of the respondents (*n* = 10,243)

Variables	Number	Percentage (%)
Gender		
Female	5592	54.6
Male	4651	45.4
Age (year)		
18-	4304	42.2
45-	3688	35.9
60-	2251	21.9
Education		
Illiteracy	1208	11.8
Primary school	2549	24.9
Middle school	3293	32.1
High school	1701	16.6
Undergraduate or over	1492	14.6
Economic status		
Low-income family	7641	74.6
Not low-income family	2602	25.4
SHIs coverage		
Not covered by SHI	816	8.1
Covered by NCMS	6297	61.5
Covered by MIUR	1052	10.2
Covered by MIUW	2078	20.2
Satisfaction of SHI reimbursement ratio		
Not satisfied	1177	11.5
Uncertain	6043	59.0
Satisfied	3023	29.5
Geographical area		
Eastern area	4253	41.5
Central area	3237	31.6
Western area	2753	26.9
District		
Urban	5581	54.5
Rural	4662	45.5
General practitioners		
Inadequate	7428	72.5
Medium	2275	22.2
Adequate	540	5.3
Satisfaction of the latest treatment		
Not satisfied	1516	14.8
Uncertain	5784	56.5
Satisfied	2943	28.7
In total	10,243	100.0

*SHI Social health insurance, *NCMS* New rural cooperative medical service, *MIUR* Medical insurance for urban residents, *MIUW* Medical insurance for urban workers

### Univariate analysis of perceived equity of the health care system

Tables 2 and 3 illustrates that respondents’ overall evaluations of the equity of the Chinese health care system were generally positive (6.7 ± 2.6, 95% CI: 6.64 to 6.74). In detail, the statistical differences in terms of perceived equity among variates of gender, age, economic status, and satisfaction with the latest treatment were insignificant (*P >* 0.05). There was a significant relationship between education level and a score of perceived equity. According to the results, respondents with a higher education level were inclined to give a lower score on equity (*P <* 0.01). SHI coverage affected respondents’ perception, and the highest score of perceived equity was found among the group covered by the New Rural Cooperative Medical Scheme (NCMS, 6.80 ± 2.59, *P <* 0.05). There were no statistical differences in terms of perceived equity among groups not covered by SHI or covered by MIUR or MIUW. The score of the perceived equity of respondents satisfied with the SHI reimbursement ratio was highest compared with respondents who were not satisfied or were uncertain (*P <* 0.05). The results of the univariate analysis showed that the respondents’ perceived equity varied across different regions. In detail, from eastern to western areas, the score of perceived equity decreased accordingly (*P <* 0.05), and respondents from rural areas considered the health care system more equitable than did urban residents (6.88 ± 2.56 vs. 6.53 ± 2.65, *P <* 0.05). The perceived equity was significantly related to the number of GPs, and respondents from provinces with inadequate GPs scored lowest in terms of the equity (*P <* 0.05).

**Table 2:** Characteristics of respondents’ perceived equity of the Chinese healthcare system

Variables	The score of perceived equity (Mean ± SD, 0~10)	***P***
Gender		
Female	6.72 ± 2.59	0.271
Male	6.66 ± 2.65	
Age (year)		
18-	6.70 ± 2.61	0.277
45-	6.73 ± 2.59	
60-	6.62 ± 2.66	
Education		
Illiteracy	6.86 ± 2.59	< 0.01
Primary school	6.81 ± 2.59	
Middle school	6.69 ± 2.61	
High school	6.60 ± 2.65	
Undergraduate or over	6.47 ± 2.66	
Economic status		
Low-income family	6.69 ± 2.62	0.663
Not low-income family	6.71 ± 2.59	
SHIs coverage		
No health insurance	6.68 ± 2.57	< 0.01
NCMS	6.80 ± 2.59	
MIUR	6.48 ± 2.69	
MIUW	6.49 ± 2.67	
Satisfaction of SHI reimbursement ratio		
Not satisfied	6.57 ± 2.69	< 0.01
Uncertain	6.63 ± 2.63	
Satisfied	6.87 ± 2.56	
Geographical area		
Eastern area	6.90 ± 2.57	< 0.01
Central area	6.65 ± 2.58	
Western area	6.42 ± 2.70	
District		
Urban	6.53 ± 2.65	< 0.01
Rural	6.88 ± 2.56	
Number of GP per 10,000 residents		
Inadequate	6.65 ± 2.61	< 0.01
Medium	6.77 ± 2.61	
Adequate	6.98 ± 2.70	
Satisfaction of the latest treatment		
Not satisfied	6.66 ± 2.67	0.269
Uncertain	6.67 ± 2.61	
Satisfied	6.76 ± 2.60	
In total	6.69 ± 2.59

**Table 3:** Analysis of the associated factors of the perceived equity by linear regression

Variables	Unadjusted model		Adjusted model	
	β	95.0% C.I.	β	95.0% C.I.
Age	−0.030	−0.095~0.035	−0.132***	−0.203~ − 0.062
Education	−0.101***	−0.143~ − 0.059	−0.104***	−0.153~ − 0.056
Satisfaction of SHI reimbursement ratio	0.043***	0.023~0.063	0.044***	0.024~0.063
Geographical area	− 0.241***	− 0.303~ − 0.179	− 0.268***	− 0.338~ − 0.199
District	0.349***	0.247~0.450	0.348***	0.237~0.458
Number of GP per 10,000 residents	0.150***	0.080~0.220	0.087*	0.008~0.165
Model fit			*F* = 27.777, *P* < 0.001	

*P < 0.05,**P < 0.01,***P < 0.001

### Multivariate linear regression model results

To explore the association between respondents’ demographic characteristics and their perceived equity of the Chinese health care system further, a multivariate linear regression model was constructed. According to the modelling results, younger respondents reported a higher score of perceived equity than their elder counterparts (β = − 0.132, 95% CI: − 0.202~ − 0.061, *P <* 0.001). Respondents with lower education levels were significantly more likely to consider the Chinese health care system equitable (β = − 0.102, 95% CI: − 0.15~ − 0.054, *P <* 0.001). Satisfaction with the SHI reimbursement ratio was found to be a protective factor for perceived equity. Respondents satisfied with SHI reimbursement ratio tended to score equity higher in the survey (β = 0.179, 95% CI: 0.097~0.262, *P <* 0.001). Respondents residing in eastern China and rural areas were significantly more likely to consider the Chinese health care system equitable (β = − 0.299, 95% CI: − 0.362~ − 0.237, *P <* 0.001). Meanwhile, rural respondents reported higher scores of perceived equity than urban respondents (β = 0.334, 95% CI: 0.224~0.444, *P <* 0.001). Number of GPs per 10,000 residents was another protective factor for perceived equity. Respondents from regions with adequate GPs gave higher scores in terms of perceived equity on this survey (β = 0.107, 95% CI: 0.021~0.178, *P <* 0.001). The present study found no influence from gender, economic status, SHIs coverage, or satisfaction with the latest treatment on perceived equity.

## Discussion

### Principal findings

In the present study, we found that although respondents’ perception of the equity of the Chinese health care system was positive, the marks were only at upper-middle level (6.7 ± 2.6, 95% CI: 6.64–6.74, 0–10 scale). The finding suggested that China’s efforts to build an equitable health care system are effective, but more progress and evidence-based policies are needed. Evidence from this survey showed that the perceived equity decreased with age. The possible reason for this was that the aged populations were more likely to suffer from a variety of diseases [20]. Although the morbidity rates for our observations in different ages were not yet clear, a large survey from southern China demonstrated that the prevalence of multi-morbidity among patients increased with age significantly [21]. The high morbidity rate among the aged group might finally lead to greater perceived inequity of the Chinese health care system.

In contrast to many studies reporting that education contributed to better health equity [22], our study showed more education was associated with lower perceived equity. The between-studies variance might be because of the measurement and perspective of equity differed. Most former studies defined health equity from one aspect of the health care system, including health financing, health outcomes, health service usage, health resource allocation, and so on [6, 23]. Their conclusion that education contributed to health equity was easily understandable. For example, education level was usually related to health literacy [24] and the ability to acquire health information [25], which provided guarantees for health equity from the perspective of health outcomes. However, perceived equity in our study referred to observers’ subjective and overall perception in terms of equity in their health care system. The judgments of the respondents not only originate in their feelings about health outcomes, but also about health financing, health service usage, health resource allocation, health service accessibility, and so on. In recognition of this difference, it is easier to understand the negative association between education and the perceived equity. According to our observations, respondents with higher education levels might be more concerned with their health conditions [26] and have a higher expectation of the quality of health services [27]. Therefore, in the context of being healthier than their less-educated counterparts were, the gap between the actual health care system and their ideal health care system still might make respondents with higher education levels consider the health care system inequitable.

The results of this study revealed that perceived equity decreased from east to west across China. It was undeniable that the traditional gaps in social and economic development levels across eastern to western Chinese health care settings contributed to the perception of inequity. However, the underlying reason should be analyzed for a better understanding of the unbalanced distribution of perceived equity. Compared to the densely populated eastern regions, the population density of the vast country is declining from east to west. Along with the decrease in population density, the density of health care facilities and the accessibility of health services are gradually decreasing [28]. The poor accessibility of health services might lead to the respondents’ perception of inequity. Specifically, in the current stage, telemedicine in China is still underdeveloped [29], and the poor accessibility of health services comprises the main challenge of equity in the Chinese health care system.

**Fig. 2:**
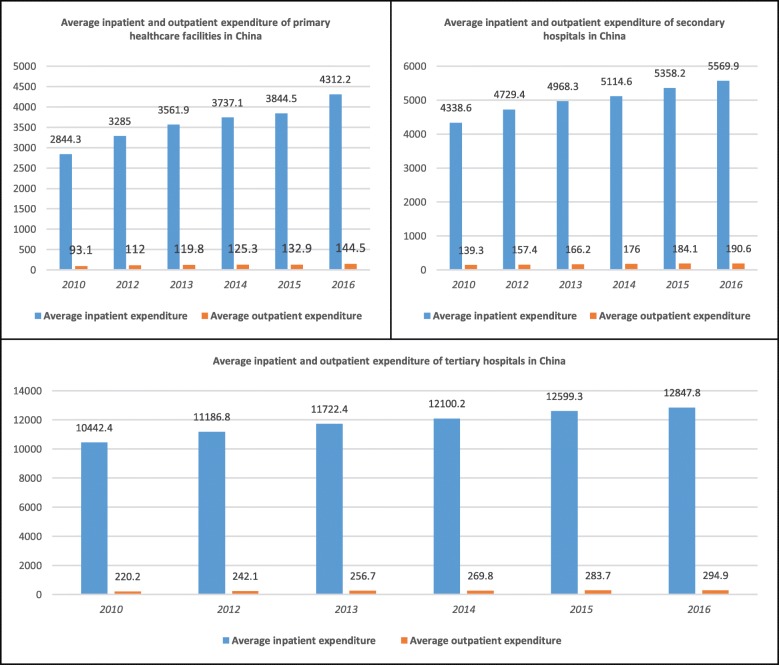
Average inpatient/outpatient expenditure (Yuan) in the Chinese health care system

We found that satisfaction with the SHI reimbursement ratio was associated with perceived equity. Since the central government decided to promote health care system reform in 1997, China has gradually established the SHIs, consisting of the Medical Insurance for Urban Workers (MIUW, since 1998), New Rural Cooperative Medical Scheme (NCMS, since 2003), and Medical Insurance for Urban Residents (MIUR, since 2007) [14]. Although the SHIs have now covered approximately 90% of China’s 1.4 billion populations, insight into its disparate framework illustrates why the SHI reimbursement ratio was associated with perceived equity. Generally, the reimbursement ratio for inpatient services (usually > 50%) is designed to be much higher than outpatient services (usually very low or 0) because the average inpatient expenditure is much higher than outpatient expenditure (see Fig. 2).

The reimbursement ratio of the insured patient with MIUW is usually higher than the ratios of NCMS and MIUR due to their high per capita financing. In addition, because social and economic development varies in different parts of China, the reimbursement ratio for the insured patients within the same health insurance system and among different health insurance systems vary by a range of 0 to > 90% according to the sufficiency of local SHI funds. From the perspective of obtaining more favourably perceived equity of the health care system, increasing SHI reimbursement ratios and integrating the various systems into a unified system should be priorities for researchers and policymakers to take forward [30].

**Fig. 3:**
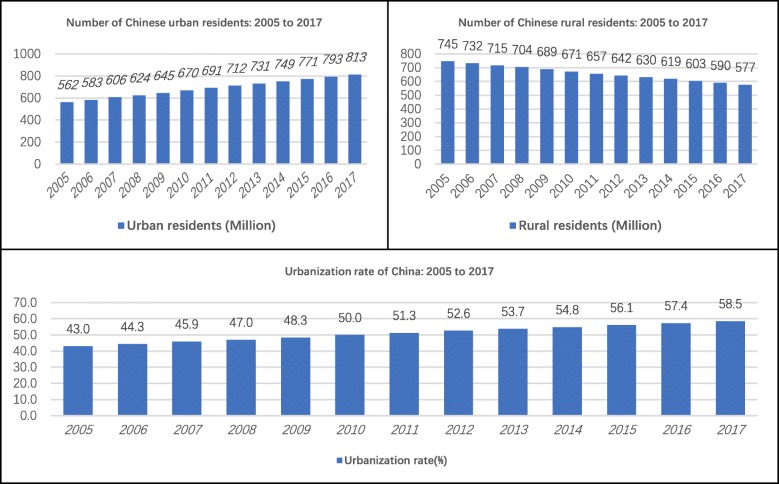
Urbanization progress in China: 2005 to 2017

The results of this study indicated that rural residents considered the Chinese health care system more equitable than urban residents did. This finding seemed contradictory to many studies, which held the view that health inequity often occurs in rural areas [31]. However, our finding is understandable given the rapid urbanization of recent years in China (see Fig. 3). From 2005 to 2017, the urbanization rate increased by 15.5%, and in 2010, the Chinese urban population began to exceed the rural population for the first time in history. However, health policy and management in many fields of the health care system failed to respond quickly and effectively to the rapid urbanization [32]. In many of the new cities, the imbalances in health financing, health service usage, and health conditions between low-income and middle-to-high-income classes, new and native citizens, and permanent and floating populations are ubiquitous [33]. In the vast rural areas, thanks to continued economic growth, the local governments are investing in more health resources to rebuild the primary-care-centred, three-level health service network [34]. Progress could be seen in the > 60% reimbursement ratio of NCMS for treatment within the counties [35], a standardized clinic for each village [36], the family doctor system [19], and the > 90% coverage rate of electronic medical records in Chinese hospitals [37]. Therefore, although rural areas were regarded as underdeveloped traditionally, the rapid progress within the rural health care system, unexpectedly but reasonably, contributed to a better perception of equity among rural residents.

This study implied that the number of GPs was likely to promote the perceived equity of the Chinese health care system. This finding could be interpreted from at least three aspects. First, a generalist, the primary care-based approach has been suggested as most appropriate for the residents, especially for the elderly with chronic diseases, because it provides continuity and coordination of care [21]. China, particularly in recent years, has paid increasing attention to the training of qualified GPs, and the quality of GP services has improved dramatically [38]. Second, the accessibility of GP services is much better than specialist services in secondary or tertiary health care facilities. According to Regional Health Planning of central and local governments in China, GP-based, 15-min health service circles are being set up in many economically developed provinces, aiming to provide primary care for the residents in 15 min [39]. Third, GP services are more affordable, which is crucial for the low-income population (in this study, the proportion of the low-income population was 74.6%.) [40, 41]. In the current stage, Chinese individuals are paying 90% for outpatient and 30% for inpatient services. The high out-of-pocket ratio is a terrible burden for the low-income population. Given the distinct average expenditures per treatment in health care facilities of different levels (see Fig. 3), respondents who used more affordable GP services, rather than expensive specialist services, were more likely to give positive comments on the equity of the health care system.

### Policy implications

Given the ongoing primary care-oriented health care reform and tiered medical service system in place in China, we propose some policy recommendations for the construction of the Chinese people’s perception of an equitable health care system based on the principal findings. From the system viewpoint, policymakers should pay attention to people’s perceived equity of the health care system, and a regular national-level survey system in terms of perceived equity of health care system should be set up. From an operational viewpoint, policymakers should focus on improving the health outcomes of the aged population through quality primary-care services; encouraging people to acquire reasonable health expectations through medical education; increasing the reimbursement ratio for medical services, especially for outpatient services; ensuring the health service usage by the low-income population through completing the family doctor system; and allocating more qualified GPs to communities. In addition, the decline of residents’ perception of equity caused by urbanization deserves the attention of health services policymakers, and a timely and integrated strategy is urgently needed.

### Strengths and weaknesses of the study

This is the first national-scale study to examine the perceived equity of the health care system and to explore its associated Chinese population. The data used in this study was gathered from a large population that had many similar characteristics to the national census population. The primary limitation of the study should be noted. Although we used data from a very large population whose characteristics were similar to the Chinese population as a whole, quite a lot of underlying information associated with perceived equity was unknown. As a cross-sectional survey, CSS 2015 was conducted to observe the overall social condition of China, and respondents’ perceived equity in the health care system was only a small part of the survey. Therefore, a national-level investigation system for rating perceived equity should be established within the health care system of China.

### Unanswered questions and future research

As a key indicator for the construction of an equitable health care system, the perceived equity deserves the attention of researchers and policymakers. The most fundamental and important approach is to develop a measurement tool for perceived equity with good reliability and validity. Moreover, research that is more theoretical should be conducted to analyze the relationship between people’s perception of the equity of the health care system and the actual equity of it.

## Conclusions

The findings of this study provided a general view of Chinese residents’ perceived equity of the health care system, demonstrated the linkages between perceived equity and part of its associated factors, and suggested policy priorities for the construction of a more equitable health care system in China. Although the overall perception of the equity of the Chinese health care system was positive, tremendous progress is still necessary. Therefore, to eliminate the sense of inequity among a range of populations, a national-level investigation system should be established within the health care system of each country. Accordingly, the survey results concerning the perceived equity by different groups should be fully considered in health policy design and health care reform.

## Data Availability

The data set of this article is available through the email of the corresponding author or at http://css.cssn.cn/css_sy/zlysj/lnsj/.

## Abbreviations

CSS: Chinese Social Survey
GP: General Practitioner
MIUR: Medical Insurance for Urban Residents
MIUW: Medical Insurance for Urban Workers
NCMS: New Rural Cooperative Medical Scheme
SHI: Social Health Insurance
SHIs: Social Health Insurance System
